# Health Problems of Students during Distance Learning in Central and Eastern Europe: A Cross-Sectional Study of Poland and Ukraine

**DOI:** 10.3390/ijerph191610074

**Published:** 2022-08-15

**Authors:** Piotr Długosz, Damian Liszka, Anastasiia Bastrakova, Luydmila Yuzva

**Affiliations:** 1 Faculty of Social Sciences, Pedagogical University of Krakow, 30-084 Krakow, Poland; 2 Department of Sociology, Kyiv International Institute of Sociology, 04070 Kyiv, Ukraine; 3 Department of Sociology, Taras Shevchenko National University of Kyiv, 01033 Kyiv, Ukraine

**Keywords:** health, COVID-19 pandemic, remote education, students, CAPI survey, Poland, Ukraine

## Abstract

The COVID-19 pandemic has significantly disrupted the functioning of society. Issues of deteriorating health were among the main problems resulting from restrictions such as self-isolation, social distancing, and remote education. The aim of this research was to attempt to probe the psychophysical condition of students after more than a year of remote education. The survey method (CAPI) was used to collect the data on a representative sample of 1000 students in Poland and 1022 in Ukraine. The research sample was selected in a randomly stratified manner, taking into account such characteristics as: gender, age, and place of residence. The results of the research showed that 44% of Polish and 50% of Ukrainian students experienced health problems. The burden of remote education mainly contributed to the reduction of physical fitness. Young Poles more often paid attention to the deterioration of mental well-being, and Ukrainians to the deterioration of their physical condition. Based on the conducted analyses, it was also established that health problems appeared more often among the females, students with lower social support and with lower trust levels. The main risk factors were Internet addiction, secondary effects of the pandemic, and negative remote education experiences. Research has shown that remote education and problems that arise in students’ households during the pandemic may have significantly contributed to the deterioration of their psychophysical condition.

## 1. Introduction

The COVID-19 pandemic has affected every country in the world in various ways and has had an unprecedented influence on the lives of people around the world. In most countries, self-isolation and social distancing became important methods of preventing COVID-19 infection [1]. Countries implemented regional and national quarantine measures, namely: restrictions on movement, prohibition of mass gatherings, closure of food service establishments, gyms, children’s clubs, closure of schools and kindergartens, etc. These circumstances, which go beyond ordinary human experience, have increased people’s levels of stress, anxiety, and helplessness.

Children and teenagers found themselves in the most unfavorable conditions during the quarantine. A UNICEF report stated: “COVID-19-the biggest global crisis for children in our 75 years of activity” [2]. The report noted that during the peak period of the pandemic, more than 1.5 billion students did not attend school due to national quarantine measures. In the first year of the crisis, almost 80% of schools worldwide did not have full-time education. In addition to schooling, children were also left without activities that they were used to before the pandemic (sports, additional foreign language classes, art classes, etc.). Restricting these activities also limited contact with friends and increased feelings of social isolation [3].

Changes that have occurred during quarantine may have long-term adverse effects on children and adolescents [1,4,5,6,7,8,9,10,11,12]. Stress due to new living conditions and conflicts in families could affect the psychophysical and psycho-emotional development of children [13]. A sedentary lifestyle (children lost the opportunity for physical activity at school and out of school) and an increase in time spent in front of a screen could affect the physical health of children, as mentioned in the study of Socioinform “How the youth of Ukraine lived during COVID-19” [14]. Negative changes in the physical health of Polish adolescents were noted, such as: deterioration of general physical shape due to limited access to sports infrastructure; weight gain due to a less active lifestyle; and overeating [15].

There is a link between increased screen time and a higher risk of health complications, mental health problems, and the negative consequences of cognitive, linguistic, social, and emotional development [16]. Some studies show significant differences in the time children spend using the Internet and TV before and during a pandemic. Thus, during the pandemic, the percentage of children who spent more than 6 h a day in front of the TV or on the Internet increased. Children also spent more time playing computer games. The increase in time spent in front of a screen was more typical on weekdays than on weekends [17].

Increasing dependence on digital instruments and increasing children’s time in front of the monitor changed the duration of sleep during the COVID-19 pandemic. The results of studies have shown a decrease in the duration of sleep of children and adolescents on weekdays and weekends [17]. In turn, lack of sleep can affect emotional health, concentration, and immune function [18,19,20]. Insufficient sleep can increase the risk of cardiometabolic disease in both children and adolescents, as well as cause anxiety changes, which may be exacerbated by poor mental health during the COVID-19 pandemic [20,21].

It is worth noting that most of the research on the health effects of a pandemic focuses on the mental problems of students instead of focusing on the existing limitations of students’ physical activity during a pandemic. Our research is one of the first to show how the pandemic and remote education adversely affected student health. Moreover, it should be noted that these are the only research results carried out on representative samples of young people in Poland and Ukraine.

The aim of our research is to try to answer the question about the psychophysical condition just before the students return to stationary school education after the summer holidays of 2021 and the risk factors of existing health burdens. We assume that spending more than a year in the remote education mode led to the deterioration of the health condition of school children. The greater the burden of remote education on students and the fewer resources they have, the greater their health problems. The practical application of the research was the preparation of recommendations for educational authorities about the problems of young people, which were crucial at the stage of students returning to school in the year 2021.

## 2. Materials and Methods

### 2.1. Participants and Procedure

The research was carried out as a part of the “Identification of barriers hindering the Ukrainian and Polish students’ return to schools” project financed by the Polish National Agency for Academic Exchange NAWA. It was the first representative survey among primary and secondary school students in both countries.

The survey method was used in the research. The technique of computer-assisted personal interviews (CAPI) was used to conduct the survey. The sample for the research was selected by a randomly-stratified method, taking into account such criteria as: household income, place of residence, type of school, and school class. Interviewers came to the randomly selected respondents meeting the above criteria. The survey was carried out in students’ homes. A legal guardian was present during the research. In the end, we managed to implement correctly 1000 interviews conducted in Poland and 1022 in Ukraine. The survey was conducted just before the return to school after summer vacation in August, 2021. The research in both countries was carried out according to the same method and at the same time.

Research in Poland was carried out by Research Collective, and in Ukraine, it was carried out by the Kyiv International Institute of Sociology. An ethics committee approval was obtained in both countries.

### 2.2. Demographic Characteristics

The research took into account demographic and social variables (Table 1) such as age, sex, place of residence, social status, number of books, number of siblings, and religiosity.

### 2.3. Trust

Trust in this research is treated as one of the indicators of social capital [22,23]. The scale of trust consists of nine items, among which there is trust in teachers, parents, neighbors, and politicians. Trust was measured on a scale ranging from definitely do not trust (=1) to definitely do trust (=5). The higher the final score, the higher the level of trust the respondent has. Cronbach’s alpha in the Polish sample was 0.76, and in the Ukrainian sample, it was 0.82. The values on the confidence scale were between 13 and 45 points. The average for Polish students was 32.5 (SD = 5.6) and 33.2 (SD = 5.2) for the Ukrainian students.

### 2.4. Short Scale of Youth’s Social Support Assessment

The Short Scale of Youth’s Social Support Assessment (SSYSSA 18) was prepared by [24] and consisted of 18 items. The scale has three subscales of student, parental, and teacher support. The responses on the scale range from definitely no (=1) to definitely yes (=5). The higher the respondent’s score, the more support he/she has. In earlier studies, Cronbach’s alpha reliability was 0.85 [24]. In the present study, Cronbach’s alpha in the Polish sample was 0.90; in the Ukrainian sample, it was 0.91. The values on the scale of social support were between 34 and 90 points. The average for Polish students was 75.8 (SD = 9.3) and 72.7 (SD = 11.5) for Ukrainian students.

### 2.5. Internet Addiction Index (IUI)

The index consisted of two items: (1) How often do you find yourself using the Internet at the expense of neglecting your home and school duties? (2) How often do you feel upset or angry when you can’t use the Internet? The responses were on a scale (ranging from very often to never) for both questions. Answers often and very often had additive values in the calculation of the index. In the present study, Cronbach’s alpha in the Polish sample was 0.83; in the Ukrainian sample, it was 0.66. The higher the index value, the greater the addiction. The value of the Internet addiction index ranged from 0 to 2. The average for Polish students was 0.37 (SD = 0.68) and 1.22 (SD = 0.81) for Ukrainian students.

### 2.6. The Amount of Time Spent in Online Lessons

The variable was measured with an open-ended question, and the students entered their daily time spent in online lessons. The mean of hours spent was 5.97 (SD = 1.11) in the Polish sample and 4.23 (SD = 1.70) in the Ukrainian sample.

### 2.7. The Index of Negative Assessment of Remote Education (INOZE)

The index was constructed by combining the answers to two questions on the evaluation of remote education. The first question was whether remote education in 2021 was a positive or negative experience for the student. The second question concerned the assessment of the level of school activities during distance education. The answer of remote education being a negative experience to the first question and answers of school activities being at a lower or much lower level for the second question had additive values in the calculation of the index. The value of the index ranged from 0 to 2. The mean value was 1.10 (SD = 0.80) in the Polish sample, and 1.05 (SD = 0.86) in the Ukrainian sample.

### 2.8. Index of Negative Impact of Remote Education (INWZE)

The prepared index consisted of 11 items describing negative experiences that occurred during remote education. They included the following responses: (1) During distance learning, my contact with teachers deteriorated, (2) During distance learning, my contact with colleagues deteriorated, (3) I could not fully understand the material of the online lessons, (4) During online lessons it was difficult to focus on what the teachers were saying, (5) I could not fully participate in the lesson online due to health problems, (6) During remote learning, other students listened to my statements and it made me embarrassed, (7) I spent too much time at the computer/laptop/tablet during remote learning, (8) I was exhausted and mentally exhausted during remote learning, (9) I could learn more while studying at school than at home, (10) During remote learning, you can download answers and use the help of parents and siblings, and (11) I’m used to remote learning and I don’t want to go back to school. The responses were marked on a Likert-type scale from strongly agree to strongly disagree. Answers of “rather agree” and “strongly agree” had additive values in the index calculation. The value of the index ranged from 0 to 11. The mean value was 4.95 (SD = 2.61) for Polish students and 3.84 (SD = 1.90) for Ukrainian students.

### 2.9. Pandemic Negative Effects Index (INSP)

In this study, there was an attempt to define the social context that may lead to overburdening and disturbing the functioning of students’ families during a pandemic (Table 2). By means of a dichotomous question (yes = 1, no = 0), students were asked whether the following events occurred in their families during the pandemic: (1) one of the parents lost their job, (2) one of the parents switched to remote work, (3) family income decreased and it was necessary to save money, (4) the possibility of eating meals deteriorated, (5) misunderstandings and conflicts arose at home, (6) contacts with peers deteriorated, (7) someone in the family was seriously ill, (8) someone in the family died of COVID-19, (9) parents broke up or divorced. The higher the INSP value, the more losses the students’ families suffered. The index value ranged from 0 to 6. For Poland, the mean was 75 (SD = 1.08), and for Ukraine, the mean was 0.81 (SD = 1.03).

### 2.10. Health loss index (ISZ)

Health losses (Table 3) were measured using the nominal scale question (yes = 1, no = 0). Students were asked whether the following situations occurred: (1) deterioration of health, (2) deterioration of mental well-being, (3) deterioration of eyesight, (4) overweightness, (5) decrease in physical fitness, and (6) deterioration of overall functioning. The higher the ISZ value, the higher the health losses the respondent experienced. The index value ranged from 0 to 6. The mean for Poland was 1.12 (SD = 1.63), and the mean for Ukraine was 1.15 (SD = 1.52).

### 2.11. Statistical Analysis

All of the analyses were performed using SPSS 25 for Windows (IBM, Chicago, IL, USA). Descriptive analysis included means and standard deviations. The Pearson correlation coefficients were used to determine the bivariate correlation. Hierarchical multiple regression analyses enabled estimation of the incremental variance explained by predictor variables on demo independent variables and those related to student workload during distance education.

## 3. Results

The research results showed there were significant changes during the pandemic in about half of the households of the students in Poland and Ukraine that participated in the survey (Table 2). In Poland, students most often reported that their parents had switched to remote work and that the students’ relations with peers had deteriorated. Meanwhile, in Ukraine, parents were more likely to lose their jobs.

The collected information on various negative events in the families of students is important because it shows the context in which students and their families functioned during the pandemic. It is assumed that the more negative changes occur in the student’s environment, the greater the health losses.

The health problems caused by the pandemic and remote education are listed in Table 3. The psychophysical condition of the students suffered the most, as indicated by the data. At the same time, it is worth adding that Polish students complained more often about the deterioration of their mental health, and Ukrainian students noticed worse physical well-being. The long period of remaining at home and spending hours in front of the computer left their mark on the students.

The results presented in Table 4 show which of the independent variables have an impact on the health of Polish students. As expected, it was observed that among Polish students, the deterioration of the general health condition was associated with the negative effects of the pandemic on their families, addiction to the Internet, negative assessment of remote education, and the negative impact of remote education on the school functioning of students. Health burden was slightly less correlated with cultural capital determined by the number of books, the level of trust, and social support.

Similar trends were observed in Ukraine (Table 5). Health burden was correlated more strongly with the index of negative effects of a pandemic, Internet addiction, social support, and the level of trust. As in the case of Polish students, it can also be seen that health losses are associated with a negative assessment of remote education, its negative impact on school functioning, and the length of time spent in front of a computer screen. Students residing in cities and girls performed a bit worse.

The results of the regression analysis appear in Table 6. Hierarchical regression analysis was used to study the relationship between socio-demographic and context variables and the health burden level. The table shows that the risk factors are searched for by regression analysis in three steps. The first model examines the impact of demosocial variables on the emergence of health burdens, the second model also includes variables that make up social capital, and the third model includes variables showing the impact of remote education and the social context of the pandemic.

In the first step, demosocial variables were introduced. The analyses showed that demosocial factors explain the variance of the dependent variable to a small extent (3%). In both groups of students, girls turned out to have more health losses. Among Polish students, greater health losses occurred in students with lower status and higher cultural capital. In Ukraine, higher health burdens appeared among students living in the city.

The introduction of social capital variables to the model in the second step resulted in a significant change in the Polish sample, and delta corrected R^2^ = 0.016; F change (3.624) = 3.552, *p* = 0.015), and indicated that the introduction of those variables had increased the level of explained variance to 4%). Sex and the number of books variables maintained their strong influence. From the newly introduced variables, social support turned out to be statistically significant.

In the Ukrainian sample, the delta corrected was R^2^ = 0.051; F change (3.953) = 15.543, *p* = 0.000), and the introduction of social capital variables increased the level of explained variance to 7%). Gender and place of residence maintained their influence. From the new variables, social support and trust turned out to be statistically significant.

In the third step, contextual variables were introduced, and the level of explained variance was 20%. In the Polish sample-delta corrected R^2^ = 0.195; F change (5.619) = 32.233, *p* = 0.000), and the introduction of those variables has increased the level of explained variance to 23%). In this model, the number of books variable maintained its impact on the level of explained variance. Among the newly introduced variables, the Internet addiction index (IUI), the remote education negative assessment index (INOZE), the remote education negative impact index (INWZE), and the pandemic negative impact index (INSP) were of significant importance.

In the Ukrainian sample, delta corrected R^2^ = 0.122; F change (5.948) = 28.959, *p* = 0.000), and the introduction of contextual variables has increased the level of explained variance to 19%). The gender, trust, and social support variables continued to be statistically significant. All variables showing the context of social and school functioning turned out to be statistically significant. Thus, the procedure of increasing the level of prediction by means of the secondary effects of the pandemic can be successful in both cases. The conclusion is that the health losses were to a greater extent determined by the nuisance of the pandemic and the negative effects of remote education.

## 4. Discussion

Before we interpret the results, it should be noted that Ukraine is much poorer than Poland. Even before the outbreak of the war in Ukraine, on 24 February 2022, the 2019 HDI (Human Development Index) rank for Poland was 32, whereas Ukraine was ranked 88 [25]. The economic situation has worsened in the poorer country, which is in line with other observations that the pandemic will increase economic and social inequalities in the world [26].

The European tradition of health education and promotion of a healthy lifestyle among young people, as indicated in medical and philosophical books [27], dates back to the 4–6th century BC, originating in ancient Greece and Rome [28]. The ancient Greeks in the 4–6th century BC developed metaphysical concepts of health based on the balance between the internal and external environment of a person and considered the influence of social and physical factors on the health of an individual [29]. This was proceeded by the development of the philosophy of sport and physical activity among students [28,30], which was aimed at promoting a healthy lifestyle and understanding its need, value, and importance [31]. Physical education is not to be seen as an additional subject that is only needed to refresh the mind before theoretical classes but should focus on the learner’s abilities and provide an environment in which learners can discover and develop their individual potential to stay motivated while working in a group and allow the learner remain confident and competent enough to engage in physical activity for a lifetime [32]. The implementation of these assumptions was based on activities undertaken since ancient times as part of personal meetings with the trainer and other students. COVID-19 and the related security measures that were implemented to limit its spread (remote learning, self-isolation, lockdown) limited or even temporarily prevented the implementation of the basic assumptions of sports philosophy in 2020–2022 in Central and Eastern Europe.

The implementation of remote education for students and “home-office” for their parents and guardians, both methods proposed by governments to ensure safety during the COVID-19 pandemic [33,34,35,36], disrupted the interaction of young people with other students, their teachers, caused an imbalance between work and family life of their parents and guardians [37], and had other negative effects for families, such as increasing the number of domestic conflicts or increasing the incidence of domestic violence [38,39]. In addition, the pandemic affected household finances, including those in Europe [40]. The results of our research indicated that the negative effects of the pandemic also affected the families of our respondents. A total of 42% of respondents in Poland and about half of those surveyed in Ukraine have experienced the impact of the pandemic.

Based on the results of numerous studies conducted around the world, we know that the COVID-19 pandemic had a negative impact on the mental health of students [41,42,43] and the mental health of adolescents [44,45], especially in the initial periods of the pandemic [46]. Moreover, young adults, students, and women may be among the groups most vulnerable to the impact of the COVID-19 pandemic in terms of their mental health risk [45,47,48]. Among the respondents to our study, 20% of people from Poland and 13% from Ukraine declared that their mental health deteriorated.

Regular physical activity is widely accepted as an effective preventive measure for a variety of health risk factors across all age, gender, ethnic and socioeconomic subgroups [49,50,51,52,53,54,55]. A growing amount of evidence suggests that, regardless of the level of physical activity, a sedentary lifestyle is associated with an increased risk of cardio-metabolic disease, all-cause mortality, and a variety of physiological problems [56,57]. In our research, as many as 37% of respondents from Poland and 30% from Ukraine reported a deterioration of physical health, 21% of students from Poland and 13% from Ukraine reported a deterioration in overall functioning, and problems with being overweight were reported by 18% of respondents from Poland and 16% of respondents from Ukraine.

Evidence of the negative impact of the COVID-19 pandemic on the physical activity of pupils and students can be found in numerous studies [58,59,60] describing the risk factors for the health of people in self-isolation or distance learning. Noteworthy is the meta-analysis which showed a significant increase in body weight and BMI during lockdown among children and school-age adolescents [61]. Furthermore, previous studies of children and adolescents aged 5 to 17 show that a sedentary lifestyle (assessed mainly by watching TV) for more than 2 h a day was associated with reduced performance, lowered self-esteem, reduced pro-social behavior, and lowered educational achievement of children and adolescents of school age [62].

We found that among students, the deterioration in general health was related to the negative effects of the pandemic in the family, Internet addiction, negative assessment of remote education, and the negative impact of remote education on students’ school functioning. Previous research results indicate that stress factors associated with negative life situations [63,64,65] or with the demands placed on students during school education [66] affect the general health of a person experiencing these stressors. Stress itself can have an impact on physical activity, causing exercise to be abandoned or reduced [67]. For our respondents, such stressors could be events that occurred in the family during the pandemic, problems related to the implementation of remote education, or new educational challenges.

Our research also confirmed the results of previous studies [68,69], which showed a relationship between students’ addiction to the Internet and their health problems. These problems may include, inter alia, chronic fatigue, the onset of anxiety, vision problems, weight disturbances [70], and sleep problems [71]. In our research, 20% of respondents from Poland and 26% from Ukraine admitted that they often or very often neglected their duties because they were using the Internet.

In our research, variables such as trust and social support (teachers, parents, peers) turned out to be statistically significant. This also confirms the results of previous studies, which showed that teacher-student relationships characterized by trust protected adolescents aged 13–18 from depression and misbehavior [72] and that there are links between trust in parents and peers and adolescent health [73,74], including students with disabilities [75].

Both small research teams and global corporations are studying the health problems of children caused by the pandemic period, namely the involvement of children in distance education and periods of long-term social isolation. In its key report, UNICEF warns that children and young people may experience the effects of COVID-19 on their mental health and well-being for many years to come [76]. Such research is urgent because in the XXI century, along with environmental problems and social cataclysms, the likelihood of new pandemics is growing. Mankind must promptly develop clear mechanisms for living in such periods (filled with remote forms of cooperation and social isolation) with minimal losses for the socio-mental state, especially of the younger generations.

### Limitations

Some limitations of the study need to be highlighted. First, our focus is on only two European countries (Poland and Ukraine), so if the results can be generalized to other Central European countries remains to be established. Furthermore, the interview was planned to last a certain amount of time. Since the respondents were students (adolescents), so we had to choose selected and limited items and scales in the questionnaire. Another limitation is that our study was performed in August of 2021. At that time, the respondents (students) were having summer vacations in both countries, so at least some health losses may not be connected with the time of remote education but rather with lack of activity during the free time students had in July of 2021. A possible bias was the mono-method used. Future research may integrate the quantitative data gained with qualitative data, such as in-depth interviews, to deepen our findings.

## 5. Conclusions

According to the UN report, vulnerabilities have been exacerbated by COVID-19 and pose a risk of a “quarantine generation”, meaning young people whose health and other aspects of life are strongly affected by the pandemic. Not only health-related ones, as in the case of patients with co-morbidities, such as diabetes, hypertension, or any respiratory disease that are especially in danger when infected with SARS-CoV-19 [77], but also different spheres that concern young people, such as the long transition from education to employment, higher unemployment, the closure of SMEs owned by young people, social disunity and marginalization of young people, especially those living within conflict regions of Ukraine [78]. That is, we are talking about economic aspects in the pandemic period, but the connection between the economic situation and health is indisputable, says Olga Kuharuk [79]. In this study, Polish students were most likely to report that their parents had moved to remote work and worsened relationships with peers, while Ukrainian students were more likely to report that their parents were losing their jobs. Remote work leads to prolonged stay of family members in one space, which often leads to increased conflict and stress. Stress causes most disorders such as anxiety, fear of recurrence, exhaustion, burnout, overexertion, and chronic fatigue, which are disorders of mental health. This mix of social and economic factors has certainly affected the health of students and their families.

It is worth noting that apart from the form of remote activity such as remote education, the COVID-19 pandemic led to the popularization of other services based on distance assistance, such as telemedicine, especially in North America and Europe [80], that, despite all the benefits it gathers, is also connected with selected problems, such as lack of legal regulations or failures of correct diagnoses in some circumstances [81].

Nonetheless, it should be kept in mind that the pandemic plunged society into specific conditions that could not be quickly analyzed to adapt properly. Furthermore, given the peculiarities of the modern information society, the impact on health (especially mental and psychological) was not only due to the spread of the virus but also through information. These circumstances indicate that in further research, it is important to apply the strategy of mixed mode and strengthen the methodology using qualitative methods.

The main conclusion, according to the results, is that the psychophysical condition of students suffered the most. It should be added that Polish students more often complained of deteriorating mental health, and Ukrainian students noticed a deterioration in physical well-being. This may be due to differences in living in European and post-Soviet countries. It is the norm for people in European countries to monitor not only physical but also mental health and, if necessary, turn to psychologists, whereas in the post-Soviet countries, only now is the practice of visiting psychologists becoming more common because in the Soviet Union such a practice was considered shameful.

## Figures and Tables

**Table 1:** Demosocial features of the studied community.

		Poland, *n* = 1000	Ukraine, *n* = 1022
Age	Mean (SD) Min–Max	M = 14.65, SD = 2.35 10–19	M = 13.46, SD = 2.34 10–18
Sex% (*n*)	Female	50 (502)	49 (496)
	Male	50 (498)	51 (526)
Place of residence% (*n*)	Village	41 (414)	32 (331)
	City	59 (586)	68 (691)
Social status	Low	21 (197)	23 (219)
	Medium	66 (611)	60 (574)
	High	12 (115)	17 (170)
Number of books	0–25	31 (304)	33 (334)
	26–200	62 (607)	61 (623)
	Over 200	6 (59)	6 (65)
Do you have siblings?	Yes	68 (682)	54 (548)
	No	32 (318)	46 (474)
Number of children in family	Mean (SD) Min–Max	M = 1.9, SD = 0.84 1–6	M = 1.7, SD = 0.98 1–10
Religiosity	Deep Believer	2 (15)	4 (45)
	Believer	67 (666)	74 (760)
	Undecided	22 (218)	13 (133)
	Unbeliever	10 (102)	8 (85)

**Table 2:** Percentage (%) and frequency (N) of answers to questions regarding the negative effects of the pandemic.

	Poland	Ukraine
The parent lost his/her job	4 (36)	9 (91)
Parents switched to remote work	18 (177)	7 (73)
Family income fell and it was necessary to save	12 (119)	35 (358)
Meal options deteriorated	2 (21)	8 (78)
Disagreements and conflicts arose at home	12 (123)	8 (79)
Contacts with peers deteriorated	15 (148)	6 (65)
Someone in the family was seriously ill	10 (95)	7 (68)
Someone in the family died from COVID-19	3 (30)	1 (9)
Parents broke up or divorced	1 (5)	1 (7)
None of the above events occurred	58 (578)	51 (516)

**Table 3:** Percentage (%) and frequency (N) of answers to questions regarding health losses.

	Poland	Ukraine
Deterioration of health	7 (70)	16 (158)
Mental well-being deterioration	20 (204)	13 (128)
Deterioration of eyesight	16 (160)	22 (221)
Overweightness	18 (176)	16 (164)
Lowering physical fitness	30 (302)	37 (373)
Deterioration of overall functioning	21 (210)	13 (136)

**Table 4:** Correlation coefficients among all predictors-Poland.

Variables	1	2	3	4	5	6	7	8	9	10	11	12	13	14
1. Sex														
2. Age	0.00													
3. Place of residence	0.01	0.00												
4. Social status	0.07 *	−0.01	0.23 **											
5. Number of children in the family	0.01	−0.02	0.07 *	0.00										
6. Number of books	0.06 *	0.03	0.08 **	0.27 **	0.02									
7. Trust	0.00	−0.16 **	−0.15 **	−0.07 *	0.00	−0.08 *								
8. Religiousness	−0.09 **	0.21 **	0.11 **	−0.04	0.02	0.10 **	−0.24 **							
9. Social support	0.11 **	−0.09	0.08 **	0.21 **	0.03	0.04	0.05	−0.21 **						
10. Internet Addiction Index (IUI)	−0.13 **	0.02	0.00	−0.06	0.01	0.00	−0.18 **	0.09 **	−0.25 **					
11. Amount of time spent in online lessons	0.09 *	0.28 **	0.03	0.04	−0.08 *	0.08 **	0.04	0.01	0.01	0.01				
12. Index of negative evaluation of remote education (INOZE)	0.15 **	0.02	0.04	0.02	0.06 *	0.05	−0.13 **	0.03	0.00	0.09 *	0.02			
13. Index of negative impact of remote education (INWZE)	0.05	−0.08 **	0.11 **	0.03	0.16 **	0.08 **	0.02	0.00	0.06 *	0.10 **	0.04	0.26 **		
14. Pandemic Negative Effects Index (INSP)	0.00	0.03	0.11 **	0.00	0.11 **	0.11 **	−0.15 **	0.11 **	−0.17 **	0.24 **	0.02	0.12 **	0.12 **	
15. Health loss index (ISZ)	0.04	0.01	0.00	0.03	0.07 *	0.13 **	−0.17 **	0.08 **	−0.10 **	0.35 **	0.09 **	0.27 **	0.25 **	0.33 **

* *p* ≤ 0.01, ** *p* ≤ 0.05.

**Table 5:** Correlation coefficients among all predictors –Ukraine.

Variables	1	2	3	4	5	6	7	8	9	10	11	12	13	14
1. Sex														
2. Age	0.00													
3. Place of residence	0.00	0.00												
4. Social status	0.01	0.03	−0.24 **											
5. Number of children in the family	0.04	−0.08 *	0.14 **	−0.17 **										
6. Number of books	0.02	0.01	0.03	0.21 **	0.02									
7. Trust	0.02	−0.17 **	0.05	0.00	0.01	0.02								
8. Religiousness	0.02	0.02	−0.16 **	0.01	−0.12	−0.16 **	0							
9. Social support	0.05	−0.15 **	0.00	0.02	0.00	0.08 **	0.65 **	−0.07						
10. Internet Addiction Index (IUI)	0.01	0.02	0.03	0.03	0.02	0.03	−0.12 **	0.02	−0.13 **					
11. Amount of time spent in online lessons	0.09	0.23 **	−0.17	0.17 **	−0.10 **	0.10 **	−0.07 *	0.00	−0.06 *	0.06 **				
12. Index of negative evaluation of remote education (INOZE)	0.05	0.05	0.03	0.06	0.02	0.02	0.01	0.02	0.03	0.04	0.02			
13. Index of negative impact of remote education (INWZE)	0.06	−0.14 **	0.03	−0.09 *	0.06 *	0.00	0.00	0.04	0.06 *	0.07 *	0.04	0.18 **		
14. Pandemic Negative Effects Index (INSP)	0.00	0.00	−0.12 **	0.02	0.03	0.06 *	−0.14 **	0.01	−0.16 **	0.13 **	0.11 **	0.06 *	0.18 **	
15. Health loss index (ISZ)	0.13 **	0.00	−0.08 **	0.04	0.04	0.04	−0.20**	0.05	−0.20**	0.21 **	0.17 **	0.13 **	0.17 **	0.22 **

* *p* ≤ 0.01, ** *p* ≤ 0.05.

**Table 6:** Results of multiple regression analyzes predicting the health burdens during remote education.

Variables	Model 1				Model 2				Model 3			
	Poland		Ukraine		Poland		Ukraine		Poland		Ukraine	
	β	*p*	β	*p*	β	*p*	β	*p*	β	*p*	β	*p*
Sex	0.077	0.050	0.141	0.000	0.100	0.013	0.150	0.000	0.081	0.029	0.123	0.000
Age	−0.009	0.823	0.002	0.956	−0.026	0.523	0.032	0.306	−0.017	0.661	−0.040	0.197
Place of residence	0.061	0.133	−0.076	0.018	−0.050	0.227	−0.082	0.013	−0.017	0.653	0.030	0.321
Social status	−0.086	0.050	0.017	0.612	−0.050	0.224	0.020	0.555	−0.037	0.352	0.023	0.465
Number of children in the family	0.011	0.791	−0.039	0.236	0.014	0.728	0.044	0.168	0.029	0.428	−0.048	−0.114
Number of books	0.170	0.000	0.032	0.325	0.154	0.000	−0.051	0.114	0.127	0.001	0.033	0.281
Trust					−0.002	0.972	−0.128	0.000	0.021	0.650	0.141	0.000
Religiosity					0.046	0.299	0.044	0.168	−0.007	0.862	0.051	0.090
Social support					0.117	0.015	−0.151	0.000	0.046	0.278	−0.080	0.005
Internet Addiction Index (IUI)									0.238	0.000	0.163	0.000
The amount of time spent in online lessons									0.073	0.063	0.148	000
The Index of Negative Assessment of Remote Education (INOZE)									0.145	0.000	0.114	000
Index of negative impact of remote education (INWZE)									0.200	0.000	0.117	000
Pandemic Negative Effects Index (INSP)									0.176	0.000	0.155	000
F (*p* ≤ 0.000)	4.061		3.914		14.657		5.048		9.387		17.262	
R square	0.028		0.025		0.040		0.073		0.232		0.191	
Standard error	1.489		1.480		1.424		1.489		1.480		1.324	

## Data Availability

Data available per request from the corresponding author.
